# Nonpharmacological Interventions Targeted at Delirium Risk Factors, Delivered by Trained Volunteers (Medical and Psychology Students), Reduced Need for Antipsychotic Medications and the Length of Hospital Stay in Aged Patients Admitted to an Acute Internal Medicine Ward: Pilot Study

**DOI:** 10.1155/2017/1297164

**Published:** 2017-01-10

**Authors:** Stanislaw Gorski, Karolina Piotrowicz, Krzysztof Rewiuk, Monika Halicka, Weronika Kalwak, Paulina Rybak, Tomasz Grodzicki

**Affiliations:** ^1^Department of Medical Education, Jagiellonian University Medical College, Krakow, Poland; ^2^Department of Internal Medicine and Gerontology, Jagiellonian University Medical College, Krakow, Poland; ^3^Institute of Psychology, Jagiellonian University, Krakow, Poland; ^4^Department of Health Psychology, Institute of Psychology, Jagiellonian University, Krakow, Poland

## Abstract

*Purpose*. Effectiveness of nonpharmacological multicomponent prevention delivered by trained volunteers (medical and psychology students), targeted at delirium risk factors in geriatric inpatients, was assessed at an internal medicine ward in Poland.* Patients and Methods*. Participants were recruited to intervention and control groups at the internal medicine ward (inclusion criteria: age ≥ 75, acute medical condition, basic orientation, and logical contact on admission; exclusion criteria: life expectancy < 24 hours, surgical hospitalization, isolation due to infectious disease, and discharge to other medical wards). Every day trained volunteers delivered a multicomponent standardized intervention targeted at risk factors of in-hospital complications to the intervention group. The control group, selected using a retrospective individual matching strategy (1 : 1 ratio, regarding age, gender, and time of hospitalization), received standard care.* Outcome Measures.* Hospitalization time, deaths, falls, delirium episodes, and antipsychotic prescriptions were assessed retrospectively from medical documentation.* Results*. 130 patients (38.4% males) participated in the study, with 65 in the intervention group. Antipsychotic medications were initiated less frequently in the intervention group compared to the control group. There was a trend towards a shorter hospitalization time and a not statistically significant decrease in deaths in the intervention group.* Conclusion*. Nonpharmacological multicomponent intervention targeted at delirium risk factors effectively reduced length of hospitalization and need for initiating antipsychotic treatment in elderly patients at the internal medicine ward.

## 1. Introduction

Health complications among elderly inpatients are an increasing problem worldwide, resulting in a growing need for the implementation of effective preventive strategies [1]. One of the most common in-hospital complications is delirium, which according to the fifth edition of the Diagnostic and Statistical Manual of Mental Disorders (DSM-V) is defined as “an acute and fluctuating alteration of attention and awareness that is accompanied by a change in cognition that cannot be better accounted for by a preexisting or evolving dementia, and is a direct consequence of some medical condition” [2].

The development of delirium may give rise to other adverse outcomes in individuals, such as falls, cognitive and functional decline with loss of independency, prolonged hospital stay, increased risk of death, rehospitalization, and institutionalization [3–5]. Most importantly, at least 30% to 40% of cases of delirium are potentially preventable [6, 7]. Currently, there is no rationale for pharmacological prophylaxis of delirium, whereas nonpharmacological interventions are widely supported by growing evidence from clinical trials, systematic reviews, and meta-analyses [8–10]. Delirium usually has a multifactorial etiology. There have been several modifiable risk factors of delirium identified, which can be targeted by nonpharmacological interventions. They include sensory impairments, disorientation, dehydration, malnutrition, immobilization, falls, and sleep deprivation [1].

Recognizing this evidence, comprehensive nonpharmacological intervention programs preventing delirium have been launched in several medical centers in the United States (the Hospital Elder Life Program, HELP) and spread worldwide [6, 9, 11]. Despite that, the majority of hospitals have not initiated structured delirium prevention programs yet, or their protocols have been implemented partially, with a lack of adherence to the program being recognized [1, 12].

Taking into account the encouraging results of ongoing delirium prevention programs, the nonpharmacological multicomponent volunteer-based interventional program “The Wholesome Contact” has been developed as a translation of evidence-based guidelines into practice. It was designed and introduced to prevent in-hospital complications among old patients of internal medicine ward. Medical and psychology students were recruited as volunteers providing the intervention and then trained to implement the protocol. With different approaches being considered, a prevention strategy employed in the present study was mostly inspired by the REVIVE program from Australia [13] that was based on interventions delivered by volunteers, not by the medical staff. On the other hand, recruiting students of medical professions is a novelty and may come with additional advantages.

The aim of our pilot study was to assess the effectiveness of a set of tailored interventions, targeted at specific delirium risk factors, delivered to elderly inpatients by trained volunteers. Additionally, we have determined the feasibility and safety of the forthcoming prospective project on nonpharmacological prevention of delirium. It has been hypothesized that the implementation of a structured delirium prevention program would reduce the incidence of in-hospital complications, shorten the length of hospitalization, and contribute to lower mortality rates in older hospital inpatients when compared to those in the usual care group. To the best of the authors' knowledge, this is the first project of its kind in Central-Eastern Europe.

## 2. Material and Methods

### 2.1. Participants

The participants for the study were recruited from the patients of the Department of Internal Medicine and Geriatrics of the University Hospital in Krakow, Poland, from October 2013 to May 2015. The Department of Internal Medicine and Geriatrics is a 47-bed acute care, tertiary teaching hospital that provides general medical care for part of the aged population of one of the biggest Polish cities.

The inclusion criteria were as follows: patients at the age of 75 and older, being admitted for acute conditions and transferred directly from the emergency department, who were alert at the time of admission (defined as the ability to establish verbal contact and being well-oriented in time, place, and person on admission).

The following exclusion criteria were employed: life expectancy < 24 hours, being admitted due to surgical reasons or transferred from other wards, isolation due to infectious disease, and a lack of consent. We only included the patients who were discharged home (patients discharged to the other departments were excluded from the analysis, as in those patients the length of hospitalization, one of our primary endpoints, may be influenced by some alternative, nonmedical reasons, like waiting for a place in a care home). The criteria were applied to both the intervention and the control group. All medical data was obtained from the patients' medical records retrospectively.

### 2.2. Intervention Group

The patients who fulfilled the criteria and agreed to participate in the study were included in a standardized multicomponent intervention. The intervention was delivered daily for 5 initial days of the hospitalization, beginning within the first 48 hours from admission, by trained volunteers.

### 2.3. Control Group

In order to select a control group with similar characteristics to the aged patients included in the project, a retrospective individual matching strategy was used. The controls were matched to the patients in a 1 : 1 ratio, with regard to variables such as age (within five years), gender, and time of hospitalization (within 21 days). Inclusion and exclusion criteria were the same for the control group and for the intervention group. The patients included in the control group received the usual medical care that consisted of standard Evidence-Based Medicine (EBM) guided treatment of acute conditions. Additionally, each patient received physiotherapy, nutritional counseling, and psychological and social support individually, if needed.

### 2.4. Recruitment and Training of Volunteers

The group of eighteen volunteers was recruited from medical and psychology students at the Jagiellonian University in Krakow. The recruitment process started with posters and social media announcements within the university and with an introductory meeting. It was necessary for a student to have at least the first year of his/her studies completed and to sign the consent in order to be a volunteer. Since active participation in the program was a great opportunity for the volunteers to experience and practice contact with aged patients in the context of a hospital setting and to train their competences and soft skills in the interdisciplinary teams, we met a sufficient interest at the stage of recruitment.

All the volunteers participated in a structured training period (12 hours), covering theoretical knowledge and practical skills necessary to provide the intervention, before they undertook the first contact with patient. The education concerned delirium (definition, diagnosis, etiology, risk factors, treatment, and prevention) and detailed protocol of the multifactorial intervention. Volunteers were instructed about the importance of adhering to the protocol. Finally, they were informed about hospital ward organization rules, health and safety, and ethical issues. They had many opportunities to address any initial questions and concerns.

The Volunteer-Patient Contact Form was introduced to the volunteers as a tool to support and to ensure adherence to the protocol (see Supplementary Material 1 available online at https://doi.org/10.1155/2017/1297164). The Form listed all risk factors addressed by the intervention and some possible ways to manage them. Volunteers followed the list and reported on the actions aimed at the risk factors every time they met a patient.

The requirements expressed in the consent included the following: minimum one semester of expected activity in the project, taking care of at least one patient a month, providing regular reports and feedback, participation in and contribution to obligatory monthly meetings, and commitment to implementation of health and safety and ethical rules of the program. The minimum length of the activity in the project (1 semester) with a minimum level of involvement (visiting 1 patient a month) was established to ensure a balance between resources invested and effects acceptable both for volunteers and for coordinators.

The aim of the regular reporting and commitment of participation in two-hour monthly meetings of supervision and training was to maintain skills and adherence to the intervention protocol. The meetings were led by a medical doctor and a psychologist to support the volunteers and to avoid overwhelming burden of care and dropouts from the program. Additionally, single training sessions were delivered by a nurse, a physiotherapist, and a social worker in order to address all the important issues concerning the hospitalization of elderly patients. Through the whole period of the project the volunteers were coordinated, supervised, and supported by an interdisciplinary team of health-care professionals employed at the Jagiellonian University. A practicing psychologist (WK) was involved in the project, worked as a supervisor, and was accessible on call when needed. The opportunity to acquire medical knowledge and experience useful for their future profession worked as an additional reward and source of motivation for the volunteers. The volunteers were as follows: eight medical students (two from the 2nd year, four from the 3rd year, and two students from the 4th year of 6-year program) and ten psychology students (four from the 2nd year, three from the 3rd year, and four from the 4th year of 5-year psychology studies).

### 2.5. Course of the Intervention

The volunteers visited patients in pairs to support each other and to minimize the workload and the burden of care. Two volunteers—a medical student and a psychology student—were assigned to a single patient at a time in order to avoid patient's disorientation related to the volunteers' turnover and to enable them to establish a trusted relationship which was recognized as crucial for successful intervention.

Contact with a particular patient was initiated by the pair of volunteers during the first visit at a patient's bedside. Afterwards, they visited the patient separately (they took turns): daily, usually in the afternoon, for five days. However, they discussed the patient's condition and needs and shared the details of the visit with the partner to provide a regular, uninterrupted, and adequate care. During the visit, lasting approximately one hour (depending on the patient's needs and condition), they performed the intervention according to the protocol and afterwards reported in the Volunteer-Patient Contact Form. Volunteers were also encouraged to share their experiences and to discuss with the rest of teams and supervisors during and between their monthly meetings. Our rationale to conduct the multifactorial nonpharmacological intervention to prevent delirium during the first five days of patients' hospital stay was based on data showing that elderly patients are most vulnerable to develop delirium at that time [14, 15]. What is more, some of the interventions implemented seem to be the most helpful and profitable then (e.g., presenting spatial organization of hospital rooms and available services, providing clocks and calendars, but also hearing aids, glasses, dentures, and walking aids, discussing with patients their current situation, their needs, and concerns).

The details of a multicomponent, standardized intervention, comprising seven components, targeted at risk factors for in-hospital complications (esp. delirium) were presented in Table 1.

### 2.6. Outcomes

Four surrogate markers of in-hospital complications were analyzed as outcome measures: the length of stay (LOS), need for prescription of antipsychotic drugs during hospitalization, falls' occurrence, and in-hospital deaths. As delirium incidence was not assessed in a standardized way, by blinded investigators unaware of the study outcomes, it could not have been analyzed as a primary outcome and surrogate markers were necessary to apply. Nevertheless, we searched for the Polish words denoting the following: “delirium”, “delirious”, and/or “agitated” and/or “confusion” in patients' medical records retrospectively.

All outcomes were assessed retrospectively on the basis of patients' medical charts and doctors' and nurses' daily reports. Considered antipsychotic drugs included haloperidol, risperidone, perazine, and quetiapine. A fall was defined as an unexpected event when the patient came to the floor unintentionally. Patients' medical diagnoses were obtained from their medical records with the Charlson Comorbidity Index (CCI) and the Age-Adjusted Charlson Comorbidity Index (ACCI) being calculated [16, 17]. The pneumonia, asthma, and chronic obstructive pulmonary diseases (COPD) were analyzed together as respiratory diseases.

### 2.7. Statistical Analyses

All statistical analyses were performed with Statistica 10 (StatSoft Inc., Tulsa, USA). Continuous variables were expressed as mean (±standard deviation); categorical data was expressed as percentages. Normality of distribution was checked by the Shapiro–Wilk test. In the case of skewed distribution data were logarithmized. Continuous variables were compared using Student's* t*-test and categorical variables were compared using Chi^2^ test. A *p* value < 0.05 was considered significant.

## 3. Results

**Figure 1:**
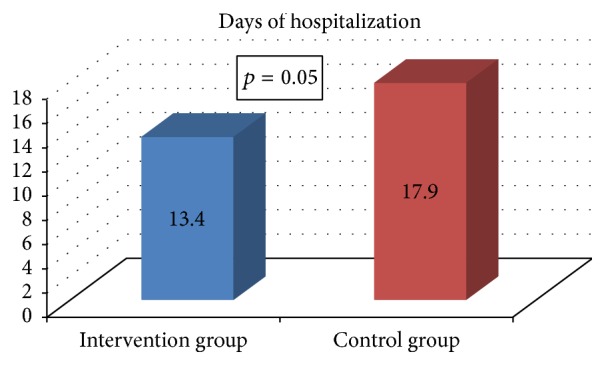
The length of hospital stay in the intervention group and the control group (median time, days).

The mean age of 130 patients included in the study was 84.7 ± 5.5 years (38.4% males). There was no difference with respect to baseline characteristics between the intervention and the control group (Table 2). The most common medical problems were listed in Table 1. The mean time of hospitalization was 15.7 ± 11.8 days. There was a trend towards a significant difference in the length of hospitalization between the intervention and the control group, mean time: 13.4 ± 7.5 versus 17.9 ± 14.4 days, respectively (*p* = 0.05) (Figure 1).

**Figure 2:**
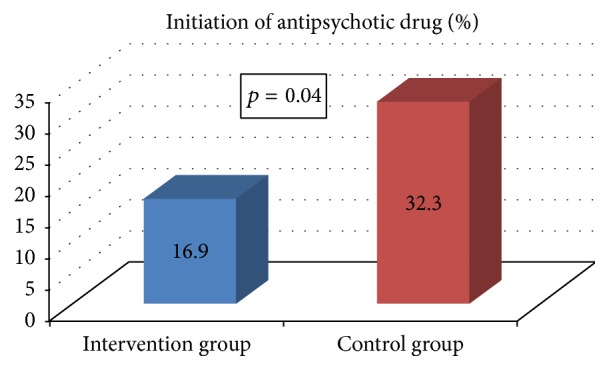
The frequency of initiation of antipsychotic medication in the intervention group and the control group.

Antipsychotic medications were initiated less frequently in those in the intervention group (16.9% versus 32.3% in the control group, *p* = 0.040) (Figure 2). There was no difference between the intervention and the control group in the number of delirium episodes reported in the patients' medical records (13.8% versus 18.5%, resp., *p* = 0.47). No statistically significant differences in the number of in-hospital deaths (3.1% in intervention group versus 10.8% in control group; *p* = 0.14) and the number of falls during hospitalization (4.61% versus 4.61%; *p* = 1.00) were noticed. No adverse effects related to patients and volunteers were reported. One of the students discontinued her participation in the project after the patient, whom she was taking care of, passed away. Prompt psychological support was offered to the student.

## 4. Discussion

Our study demonstrated that the nonpharmacological, multicomponent intervention targeted at delirium risk factors, delivered by volunteers, could be effective in reducing the length of hospital stay and the need for antipsychotic treatment initiated during hospitalization in the group of aged patients hospitalized in internal medicine ward.

The effectiveness of multicomponent nonpharmacological interventions in reducing the time of hospital stays was studied in several trials [6, 7, 13, 18–20]. However, the results are ambiguous. Hshieh et al. showed in the meta-analysis involving 3358 patients that the length of stay (LOS) tended to be shorter in the intervention group, but the difference was not statistically significant [9]. The research of Lundström et al. and Alvarez et al. proved a significant reduction in the time of hospitalization when the multicomponent intervention was implemented. The study by Lundström et al. [19] showed a significant decrease in LOS in the group of patients hospitalized in a postoperative orthopedic ward, and the trial by Alvarez et al. [20] assessed aged patients admitted to Intensive Care Unit (ICU). There was no reduction in the length of stay reported in the trials conducted in medical and geriatric wards so far [6, 7, 13, 18]. To the best of our knowledge the presented study is the first that reports a trend towards the shortening of LOS in an internal medicine ward.

Reducing LOS in the intervention group might reflect better quality of care. It may be related to a reduced number of in-hospital complications, including delirium. In the analysis conducted by Bo et al., patients who developed delirium stayed at hospital almost twice as long as those who did not experience delirium [21]. Reducing LOS is also important from an economic perspective. Cost-effectiveness of nonpharmacological interventions in delirium prophylaxis was proven in several trials [6, 13].

At the time of our pilot study, both in the intervention and in the control group, a formal diagnosis of delirium has not been carried out, which is a weakness of the study. Instead, we searched for the Polish words denoting the following: “delirium”, “delirious”, and/or “agitated” and/or “confusion” in patients' medical records retrospectively. We also decided to evaluate the need for initiation of antipsychotic medication (haloperidol, quetiapine, perazine, and risperidone) as a surrogate outcome measure for hyperactive delirium incidence. Although significant reduction in the incidences of antipsychotic drugs initiation was observerved in the intervention group, relative to the control group, this effect should be further verified in our planned prospective study, encompassing formal diagnosis of delirium. According to the obtained data, it was assumed that delirium is experienced in about 20% of patients who met the inclusion criteria for the study. These results are consistent with previously published data [22]. The number of patients hospitalized in the ward that met the criteria for inclusion was on average 12 per month. When the errors of 5% and 95% confidence interval were established then the minimal sample size required to detect the effect of reduced incidence of delirium due to intervention in the planned prospective study was estimated at 133 patients within two years.

A statistically nonsignificant reduction of mortality in the patients in the intervention group was shown. Although our preliminary outcomes need to be confirmed in a larger, randomized trial, which is planned to be performed, the results are comparable to those presented in the recent meta-analysis by Martinez et al. Martinez and colleagues pooled 3 studies with a total number of 582 patients [19, 20, 23] and a nonsignificant reduction in in-hospital mortality was observed [8]. Among these, only Vidán et al.'s study found a statistically significant increase in preventing in-hospital deaths for patients allocated to the intervention [23].

There was no difference detected in the number of falls in the intervention and in the control group. Still, results on the impact of multicomponent interventions on the number of falls in aged patients are inconclusive. Stenvall et al., in their study of 199 patients, demonstrated a significantly lower incidence of falls in the intervention group. However, the trial was performed in a different setting and with a different scope of patients when compared to our study (orthopedic ward, patients with femoral neck fracture) [24]. In the two trials carried out in the internal medicine departments only a nonsignificant trend towards lower incidence of falls was observed [13, 25].

To the best of our knowledge, our study is the first from Central and Eastern Europe to assess the impact of a nonpharmacological intervention targeted at delirium risk factors on the short-term outcomes in hospitalized older patients. What is more, it is also the first report in Europe on a trial in which trained volunteers were employed to deliver the intervention. The idea of the volunteer-based nonpharmacological intervention to prevent or treat in-hospital complications was put into action in medical settings in the USA and Australia [6, 13]. Although it is known to be challenging to recruit and train volunteers who provide high-quality care with acceptable adherence to the protocol, based on our encouraging preliminary results, we believe that the idea of involving medical and psychology students is worth considering.When developing the frameworks for the program, we had checked medical databases for research and results containing the following words: “delirium” and “volunteer” and “student”. Although the growing interest in trained volunteers being engaged in nonpharmacological prevention of delirium was visible [13, 26, 27], neither program nor initiative encompassed medical and/or paramedical students, and therefore it seems to be an innovative solution.

Apart from substantial benefits for patients, it provides a great opportunity for future medical and health-care professionals to gain valuable experience, thus creates a win-win situation. Furthermore, as mentioned above, no adverse outcomes related to patients were noted. The innovative nature of our project also covers the successful implementation of state-of-the-art research and guidelines for prevention of in-hospital complications, despite serious cultural, economic, and organizational differences being present in Poland.

We are fully aware of some weaknesses of our study. First of all, our preliminary results are based on retrospective data analysis. Secondly, there was no formal delirium assessment being performed at the time of our study. We decided to present the surrogate outcome measures (LOS, need for antipsychotic medications) instead, but at the same time detailed medical charts review was conducted. Inconsistent results obtained (higher number of those in whom antipsychotic drugs were initiated than those diagnosed with delirium) are in line with the study of van Zyl and Davidson who showed that delirium was underreported in patients' medical records [28]. Thirdly, described intervention was limited to only five days of patient's stay on the ward. The established time frame for the intervention had been based on the results of the previous delirium studies revealing that delirium incidence is the greatest on first five days of hospitalization as well as on the reasonableness and rationality for some of the familiarizing interventions [14, 15]. What is more, some of the interventions implemented seem to be the most helpful and profitable then (e.g., presenting spatial organization of hospital rooms and available services, providing clocks and calendars, but also hearing aids, glasses, dentures, and walking aids, discussing with patients their current situation, their needs, and concerns). Nevertheless, it would also be of interest to assess the effectiveness of prolonged delirium risk factors targeted intervention. Additionally, some bias may result from the individual caregivers' and proxies' involvement, as well as the fact that each patient received physiotherapy, nutritional counseling, and psychological and social support if required. As there have been few similar studies in literature published to date, we found the submitted data worth presenting, even though the results are preliminary. However, our results regarding prevention of in-hospital complications with nonpharmacological strategies seem encouraging and innovative, as well as demonstrating feasibility of the program. Further investigations are needed and planned to be continued.

## 5. Conclusion

Our pilot study supports the use of multifactorial volunteer-based nonpharmacological intervention to prevent in-hospital complications in an internal medicine ward. Medical and psychology students should be considered as potential volunteers in some other similar projects.

## Supplementary Material

The Volunteer-Patient Contact Form was designed to aid the voluteers' adherence to the intervention protocol. The form includes a list of risk factors and some suggestions and examples how they can be addressed. An open space for the voluteers' post-visit impressions allows description for any subjective evaluations of the volunteer-patient contact, encountered difficulties or concerns, and importants observations not listed in the table. The dedicated space is to be completed after each consecutive visit.

## Figures and Tables

**Table 1:** The description of a multicomponent standardized intervention.

In-hospital complications risk factor	Intervention delivered by the volunteers	Examples of actions undertaken by the volunteers
Disorientation	Strategies aimed at time, place, and situation reorientation and cognitive stimulation	Time reorientation: volunteers used calendars and clocks in order to show the current date and time Place reorientation: volunteers explained to patients spatial arrangement of the hospital Volunteers accompanied patients' walks around a ward to present spatial organization of hospital rooms and available services Situation reorientation: volunteers discussed with patients their current situation Cognitive stimulation: volunteers read newspapers loudly to the patients and discussed ongoing events with them If applicable, volunteers discussed some problems with patients' caregivers and encouraged them to perform similar intervention

Psychological distress	Strategies aimed at reducing patients' psychological distress	Volunteers discussed with patients their current situation and their needs and concerns to build trusted relationship and to obtain an insight into patients' preferences and feelings related to the ongoing hospitalization; volunteers accompanied and supported patients and arranged conversation, recreation, and relaxation If applicable, volunteers discussed some problems with patients' caregivers and encouraged them to perform similar intervention

Immobility	Strategies aimed at reducing the time of patients' immobility	Volunteers explained to patients and their caregivers potential benefits of physical activity and disadvantages of restricted mobility; volunteers encouraged and accompanied patients during their walks around a ward; if patients presented any mobility problems, volunteers assisted them with walking aids or wheelchairs or tried to mobilize them at the bedside If applicable, volunteers discussed with patients the reasons for limiting their physical activity and tried to address the obstacles If applicable, volunteers discussed some problems with patients' caregivers and encouraged them to perform similar intervention

Dehydration	Strategies aimed at improving patients' state of hydration	Volunteers explained to the patients and their caregivers all the potential benefits of adequate hydration and encouraged patients to drink more; volunteers made sure that patients had water or any beverage close at hand and accessible If needed they supported patients while drinking and provided them with sippers; if applicable, volunteers discussed some problems with patients' caregivers and encouraged them to perform similar intervention

Malnutrition	Strategies aimed at improving patients' state of nutrition	Volunteers explained to patients and their caregivers all the potential benefits of good nutrition; volunteers discussed with patients any problems related to food intake, for example, problems with chewing, swallowing, or loss of appetite, and tried to address them if possible (e.g., ask caregivers to provide patients with dentures); when needed, they assisted patients with cutting food and bringing it into the patients' mouth with fork or spoon If applicable, volunteers discussed some problems with patients' caregivers and encouraged them to perform similar intervention

Sensory deprivation	Strategies aimed at improving patients' vision and hearing	Volunteers checked whether patients had their eyeglasses and hearing aids accessible Volunteers found out whether patients needed eyeglasses or hearing aids and informed patients' family when they were needed to be delivered to hospital; volunteers provided the patients with magnifiers for reading and showed them how to use bedside lamps for reading in the evening If applicable, volunteers discussed some problems with patients' caregivers and encouraged them to perform similar intervention

Sleep problems	Strategies aimed at improving patients' sleep quality and quantity	Volunteers educated patients on the basic elements of sleep hygiene; all the patients were advised to avoid naps during the day and were asked about any sleep related problems If applicable, volunteers discussed some problems with patients' caregivers and encouraged them to perform similar intervention

**Table 2:** Baseline characteristics of the intervention and the control group.

Variables	Intervention group (*n* = 65)	Control group (*n* = 65)	*p* value
Age; mean ± SD (years)	84.9 ± 5.3	84.4 ± 5.6	0.63
*n* (%) men	25 (38.4%)	25 (38.4%)	1.00
CCI; mean ± SD (points)	3.66 ± 2.06	3.63 ± 1.75	0.93
ACCI; mean ± SD (points)	7.65 ± 2.34	7.66 ± 1.78	0.97
*n* (%) respiratory diseases	32 (49.2%)	36 (55.4%)	0.48
*n* (%) chronic heart failure	30 (46.9%)	32 (49.2%)	0.79
*n* (%) diabetes mellitus type 2	24 (36.9%)	19 (29.2%)	0.35
*n* (%) chronic kidney disease	25 (38.4%)	27 (41.5%)	0.72
*n* (%) neoplasm (all types included)	10 (15.4%)	5 (7.7%)	0.17
*n* (%) anemia	15 (23.1%)	18 (27.7%)	0.55
*n* (%) urinary tract infection	10 (15.4%)	13 (20.0%)	0.49
*n* (%) cognitive impairment	15 (23.1%)	13 (20.0%)	0.67

CCI: Charlson Comorbidity Index. ACCI: Age-Adjusted Charlson Comorbidity Index.
