# Supplementary material for: Nonpharmacological Interventions Targeted at Delirium Risk Factors, Delivered by Trained Volunteers (Medical and Psychology Students), Reduced Need for Antipsychotic Medications and the Length of Hospital Stay in Aged Patients Admitted to an Acute Internal Medicine Ward: Pilot Study
Source: Biomed Res Int. 2017 Jan 10;2017:1297164. doi: 10.1155/2017/1297164 (PMC5259647; doi:10.1155/2017/1297164)
Supplement: Supplementary file 1 — The Volunteer-Patient Contact Form was designed to aid the voluteers' adherence to the intervention protocol. The form includes a list of risk factors and some suggestions and examples how they can be addressed. An open space for the voluteers' post-visit impressions allows description for any subjective evaluations of the volunteer-patient contact, encountered difficulties or concerns, and importants observations not listed in the table. The dedicated space is to be completed after each consecutive visit. [file 1297164.f1.pdf]

Suppl.1 The Volunteer-Patient Contact Form

The Volunteer-Patient Contact Form

Patient’s name	Volunteer’s name

Is the risk factor present? Yes=1, No=0<br><br>If yes, have we managed to modify that factor? Yes=(+), No=(-)

Date / Risk factors									Intervention examples
Immobility									A short walk with the patient, simple exercises in bed.
Dehydration									Making sure that the patient has enough liquids at hand, supplying some if there are not any. Paying attention to whether the patient is able to drink by him/herself. If needed, assisting with drinking, e.g., by passing a straw.
Disorientation									Providing information about current time and place, daily routine on the ward, meals and doctors’ rounds schedule. Explaining to the patient how to operate a bed remote control and a buzzer summoning a nurse.

Hearing problems									Speaking loudly and making sure that the patient can hear well. If the patient is using a hearing aid, checking whether it is turned on and has working batteries.
Vision problems									Checking if the patient needs glasses and has them with him/her. Asking patient's family to bring the glasses if they were left at home. Offering a magnifying glass for reading.
Lack of cognitive stimulation									Chatting about the patient's interests and current affairs. Bringing a newspaper.
Sleep deprivation									In case noise is the issue, advising family on bringing earplugs for the night. Taking care of the patient's circadian rhythm – encouraging the patient to avoid sleeping during the day, so that he/she can sleep better at night (within reason).
Malnutrition									Checking whether the patient uses dentures and has them with him/her – if not, intervening to provide them. Passing any information about patient's difficulties with food intake to the hospital personnel. Assisting the patient with eating, e.g., visit during meal times.
Concealed aliments									Encouraging the patient to share his/her aliments with the personnel.
Family involvement									Tactfully encouraging the patient's family to implement the above interventions as well.

Post-visit impressions (e.g., course of the meeting, assessment of the patient, self-evaluation)
